# Alveolar Macrophages Treated With *Bacillus subtilis* Spore Protect Mice Infected With Respiratory Syncytial Virus A2

**DOI:** 10.3389/fmicb.2019.00447

**Published:** 2019-03-12

**Authors:** Ji Eun Hong, Yoon-Chul Kye, Sung-Moo Park, In Su Cheon, Hyuk Chu, Byung-Chul Park, Yeong-Min Park, Jun Chang, Jae-Ho Cho, Man Ki Song, Seung Hyun Han, Cheol-Heui Yun

**Affiliations:** ^1^Department of Agricultural Biotechnology, Research Institute of Agriculture and Life Sciences, Seoul National University, Seoul, South Korea; ^2^Center for Food and Bioconvergence, Seoul National University, Seoul, South Korea; ^3^Laboratory Science Division, Department of Molecular Vaccinology, International Vaccine Institute, Seoul, South Korea; ^4^Division of Zoonoses, Center for Immunology and Pathology, Korea Center for Disease Control and Prevention, National Institute of Health, Cheongju, South Korea; ^5^Institute of Green Bio Science and Technology, Seoul National University, Seoul, South Korea; ^6^Department of Immunology, School of Medicine, Konkuk University, Chungju, South Korea; ^7^Graduate School of Pharmaceutical Sciences, Ewha Womans University, Seoul, South Korea; ^8^Academy of Immunology and Microbiology, Institute for Basic Science, Pohang, South Korea; ^9^Department of Oral Microbiology and Immunology, DRI, and BK21 Plus Program, School of Dentistry, Seoul National University, Seoul, South Korea

**Keywords:** respiratory syncytial virus, *Bacillus subtilis*, spore, alveolar macrophage activation, MyD88

## Abstract

Respiratory syncytial virus (RSV) is a major pathogen that infects lower respiratory tract and causes a common respiratory disease. Despite serious pathological consequences with this virus, effective treatments for controlling RSV infection remain unsolved, along with poor innate immune responses induced at the initial stage of RSV infection. Such a poor innate defense mechanism against RSV leads us to study the role of alveolar macrophage (AM) that is one of the primary innate immune cell types in the respiratory tract and may contribute to protective responses against RSV infection. As an effective strategy for enhancing anti-viral function of AM, this study suggests the intranasal administration of *Bacillus subtilis* spore which induces expansion of AM in the lung with activation and enhanced production of inflammatory cytokines along with several genes associated with M1 macrophage differentiation. Such effect by spore on AM was largely dependent on TLR-MyD88 signaling and, most importantly, resulted in a profound reduction of viral titers and pathological lung injury upon RSV infection. Taken together, our results suggest a protective role of AM in RSV infection and its functional modulation by *B. subtilis* spore, which may be a useful and potential therapeutic approach against RSV.

## Introduction

Respiratory syncytial virus (RSV) causes serious disease symptoms of bronchiolitis and acute lower respiratory tract infection resulting in up to 200,000 annual deaths worldwide, especially for the infants and elderly (Nair et al., 2010). Recent study has also suggested that children younger than 6 months old suffering from RSV amounted to 1.4 million hospital admissions and 27,300 for in-hospital death (Shi et al., 2017). Despite the numerous trials, effective preventive strategies against RSV infection have been limited and turned out to be unsuccessful. This is because of the unique ability of RSV to disarm host of both innate and adaptive immunity (Meng et al., 2014) causing overall failure of host immune defense mechanisms, which is mainly due to non-structural 1 and 2 proteins. Indeed, a poor innate response has previously been well documented particularly at the initial stage of RSV infection (Marr et al., 2013) and several attempts have been made to improve this stage in RSV infection. In this regard, it has been suggested that successful protection against RSV can be mediated by activation of innate immune cells via toll-like receptor (TLR) 2 and 4 stimulation (Kurt-Jones et al., 2000; Murawski et al., 2009), but not by caspase-1 pathway (Shim and Lee, 2015).

One of the primary innate immune cell types in the lung is alveolar macrophages (AM), which play a pivotal role to maintain homeostasis and to induce effective defense mechanism. AMs have unique properties compared to other macrophages in different tissues because they have direct contact with external environment, which could allow rapid and direct recognition of antigens derived from invading pathogens or allergens followed by the initiation of immune responses (Hashimoto et al., 2011; Herold et al., 2011). Recent studies have shown that failure of early AM-mediated defense response caused insufficient protection against various viral pathogens infecting respiratory tracts and led to diminished recruitment of immune cells followed by disrupted lung homeostasis (Maus et al., 2002; Snelgrove et al., 2008). Despite the importance of AMs in various respiratory viral diseases, the precise role and protective mechanisms of AM in RSV infection are yet to be investigated.

Previous attempts using probiotics have gained an insight for the priming innate immune system where the administration of such probiotics induced a rapid activation of innate immunity and consequently enhanced protective efficacy against respiratory viral infection (Garcia-Crespo et al., 2013; Park et al., 2013; Lee et al., 2016). Furthermore, spore derived from probiotics, as we used in this study, is known to be one of the alternatives not only for inducing enhanced innate immune responses but also for safety and stability (Hong et al., 2008). *Bacillus subtilis* has been known to change its status into spore form under the harsh circumstances such as starvation. After sporulation, it can survive with resistance to heat, cold, or other enzymatic assaults (Tan and Ramamurthi, 2014). Because of their stability, several researchers have tried to use spores as probiotics or adjuvants to enhance the health and to protect against infections caused by microbes such as influenza virus (Song et al., 2012) or *Clostridium difficile* (Colenutt and Cutting, 2014) in mice and human trials (Lefevre et al., 2015, 2017).

In this study, we tried to use *B. subtilis*-derived spore to overcome RSV infection and investigated whether and how *B. subtilis*-derived spore would influence on the AMs to induce effective innate immunity and subsequently to protect host from RSV infection.

## Results

### Intranasal Delivery of *B. subtilis* Spore Enhanced Anti-viral Immunity to RSV Infection

**FIGURE 1:**
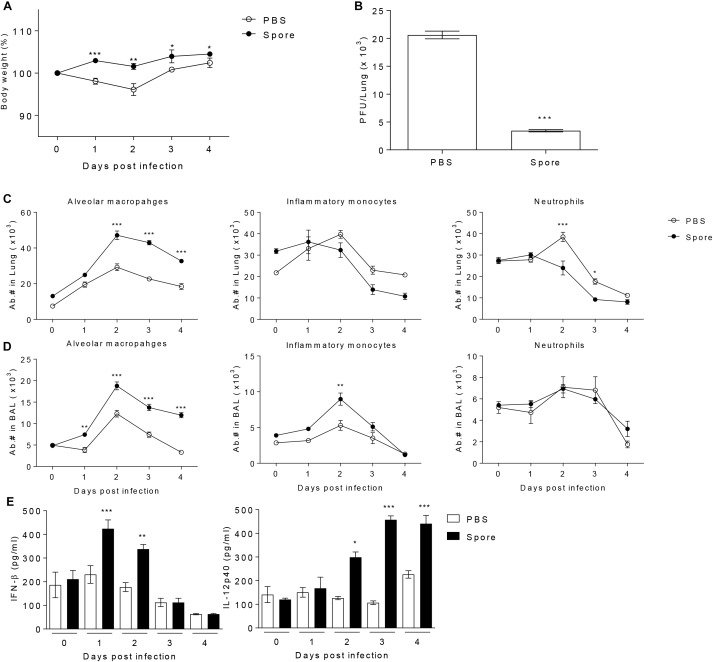
Pre-treatment with spore through intranasal route reduces the disease severity following RSV infection and induces the population change of alveolar macrophages (AM) and enhances antiviral effector molecules. Mice were administered with 1 × 10^9^ CFU of spore via i.n. route at 5 days prior to RSV infection with 2 × 10^6^ PFU per mouse (*n* = 3). **(A)** Body weight was monitored daily after the infection and **(B)** viral load in the lungs was analyzed by plaque assay at 4 days post-infection (DPI). Change of various innate immune cells in the **(C)** post-lavaged lung and **(D)** BAL fluid was analyzed by flow cytometry at 0 to 4 DPI. Empty and filled circles indicate PBS and spore pre-treated mice, respectively. **(E)** IFN-β and IL-12p40 in BAL fluid were measured by ELISA. Data are expressed as mean ± S.E.M. for the group. Significant differences from results with the PBS control are ^∗^*P* < 0.05; ^∗∗^*P* < 0.01; and ^∗∗∗^*P* < 0.001, respectively.

It has been reported that *in vivo* administration of prebiotics or probiotics such as *Lactobacillus* can facilitate protective immunity against influenza virus infection (Kiso et al., 2013; Lee et al., 2013). These previous reports prompted us to test the potential of bacterial particular substance, *B. subtilis-*derived spore to protect against RSV infection. To address this, a group of mice was administered with or without spore intranasally (i.n.) prior to RSV infection and then infected them with RSV. The effect of spore was clear in body weight loss; untreated control mice lost 10–20% total body weight with a peak reduction of day 2 post-infection (p.i.), which is well-known symptom of RSV infection in mouse model, whereas mice treated with spore did not lose their body weight (Figure 1A). It should be noted, however, that the weight loss after RSV infection appeared to be transient, which is a limitation of RSV mouse infection model, so the control mice began to gradually gain weight back to near normal level by day 4 post-infection. To support the body weight data, there was about 4–5-fold decrease in viral loads in the lung of the spore-treated mice compared to those of control mice (Figure 1B). These results suggest that delivery of *B. subtilis* spore not only prevent the pathology, but also help clearance of virus against RSV infection.

### I.n. Administration of *B. subtilis* Spore Increases the Number of Alveolar Macrophages

To identify the cellular mechanism by which *B. subtilis* spore influences on anti-viral immunity, we analyzed various innate immune cells in the lung with or without spore treatment. We found that there was the most notable increase in AM both in the lung and bronchoalveolar lavage (BAL) along with inflammatory monocytes or neutrophils (Figure 1C,D), implicating AMs might play an important role in mediating innate immunity to RSV. In line with increased numbers of AM, spore-treated mice showed increased gene expression of GM-CSF and well-known M1 macrophage-related cytokines (Murray et al., 2014) including TNF-α, IFN-γ, IL-12p40, and IL-6 (Supplementary Figure S1). The cytokine producing ability of spore was also apparent in protein level of effector cytokines such as IFN-β and IL-12p40 (Figure 1E), which are also categorized in M1 macrophage-related cytokines. These results suggest that administration of *B. subtilis* spore induced significantly enhanced number of AMs, and augmented expression of effector cytokines which are related to M1 macrophage differentiation.

### Depletion of Alveolar Macrophages Aggravates Disease Severity in Mice Infected With RSV

**FIGURE 2:**
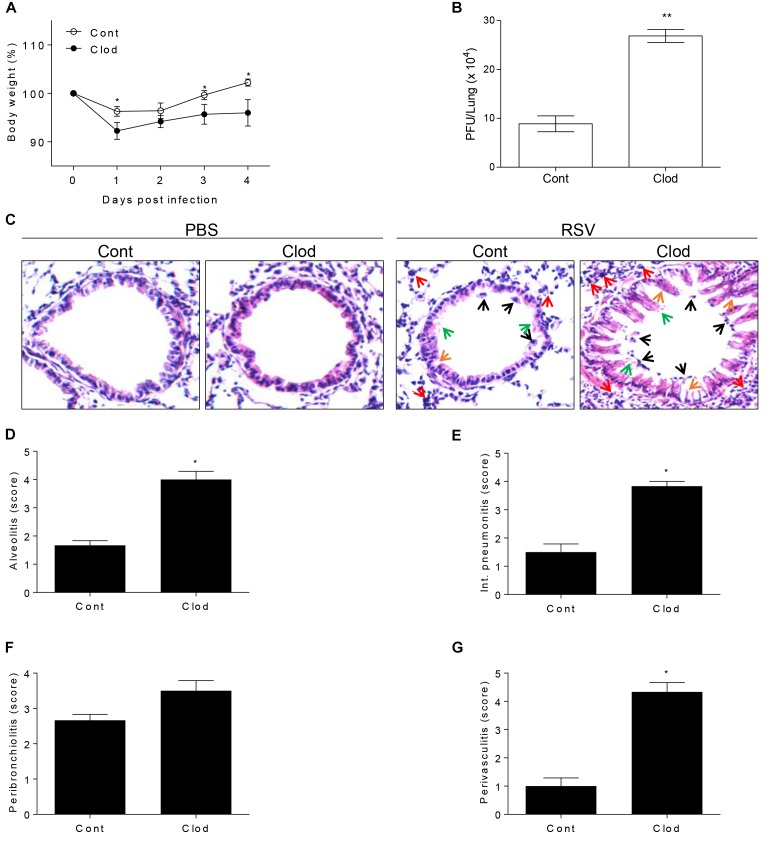
Mice selectively depleted with AMs fail to protect RSV infection. Mice were injected i.t. with clodronate-encapsulated liposome twice on days 1 and 3 prior to RSV infection. **(A)** Body weight was monitored daily after the infection and **(B)** viral load in the lungs was analyzed by plaque assay at 4 DPI (*n* = 3). At 4 DPI, perfused lungs were stained with H&E **(C)** for histological examination by microscopy at 200 × magnifications and **(D–G)** scored for histopathology. “Cont” indicates the mice injected with control liposome and “Clod” indicates the mice injected with clodronate-encapsulated liposome. Arrows indicated as follows; orange, epithelium thickness and destruction; green, pulmonary edema; red, inflammatory cells; and black, cell death. Data are expressed as mean ± S.E.M. for the group. Significant differences from results with the PBS control are ^∗^*P* < 0.05 and ^∗∗^*P* < 0.01, respectively.

In viral infection condition, it is known that AM have a critical role during the early phase of infection (Schneider et al., 2014a) because of the alarming failure and lack of scavenger activity against infected cells (Kumagai et al., 2007; Pribul et al., 2008). Moreover, AM-depleted mice are unable to deliver the antigen to dendritic cells for the antigen presentation (Ugonna et al., 2014) in order to strengthen the following adaptive immune response. To examine the particular role of AM in RSV infection, we conducted the depletion of AM by injection of clodronate-encapsulated liposome (Clod) through the intratracheal (i.t.) route. As a result, absolute number of AM in the lungs and BAL from Clod-treated mice declined by approximately 85% compared to that in control (Supplementary Figure S2) without significant histological changes (Figure 2C). At day 1 post-infection of RSV, AM-depleted mice showed a sharp decline in body weight (Figure 2A) and at day 4, they have significantly higher viral loads in lung than control (Figure 2B). Pathological results also showed the severity of infection with displayed thickened alveolar epithelium, destruction of epithelial walls, overall alveolar swelling, and the accumulation of immune cells in the lung (Figure 2C). Furthermore, the accumulation of inflammatory cells in the interstitial space, bronchi, and vessel was apparent in lungs from AM-depleted mice (Figure 2D–G). Taken together, depletion of AM at the initial phase of RSV infection leads to exacerbated disease severity coincident with increased pulmonary inflammation.

### Spore Directly Enhances the Antiviral Function of Alveolar Macrophages

**FIGURE 3:**
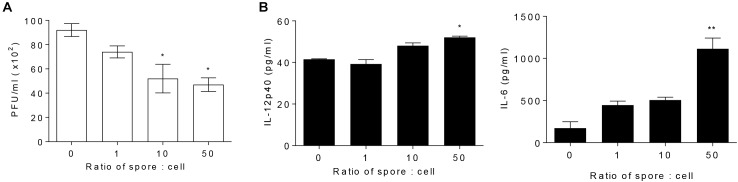
Alveolar macrophages pre-treated with spore extend their antiviral effects. MH-S cells were stimulated with spore for 24 h, with a ratio of MH-S:Spore at = 1:0, 1:1, 1:10, and 1:50. Then, the cells were washed and infected with 1 MOI RSV for 12 h. **(A)** The viral load was analyzed by using plaque assay and **(B)** the production of IL-12p40 and IL-6 was measured by ELISA. Data are expressed as mean ± S.E.M. of three replicates. ^∗^ and ^∗∗^ indicate significant differences at *P* < 0.05 and *P* < 0.01, respectively.

To explore direct role of AM in cellular level and the effect of *B. subtilis* spore, AM cell line, MH-S, was treated with spore for 24 h and then infected with RSV. The number of plaques of RSV was significantly reduced when the cells were incubated with spore in a dose-dependent manner (Figure 3A). Inflammatory cytokines, IL-12p40 and IL-6, in the supernatant from the cells treated with spore followed by RSV infection were measured as an initial assessment for their antiviral activity (Ugonna et al., 2014). The results showed that the spore treatment led to substantial increase of IL-6 and, to a lesser extent, IL-12p40 (Figure 3B). These results demonstrated that *B. subtilis* spore directly promotes the anti-viral activity of AM, especially at the early time point after the infection, in a dose-dependent manner.

### Alveolar Macrophages Activated by Spore Have a Pivotal Protective Role in Mice Infected With RSV

**FIGURE 4:**
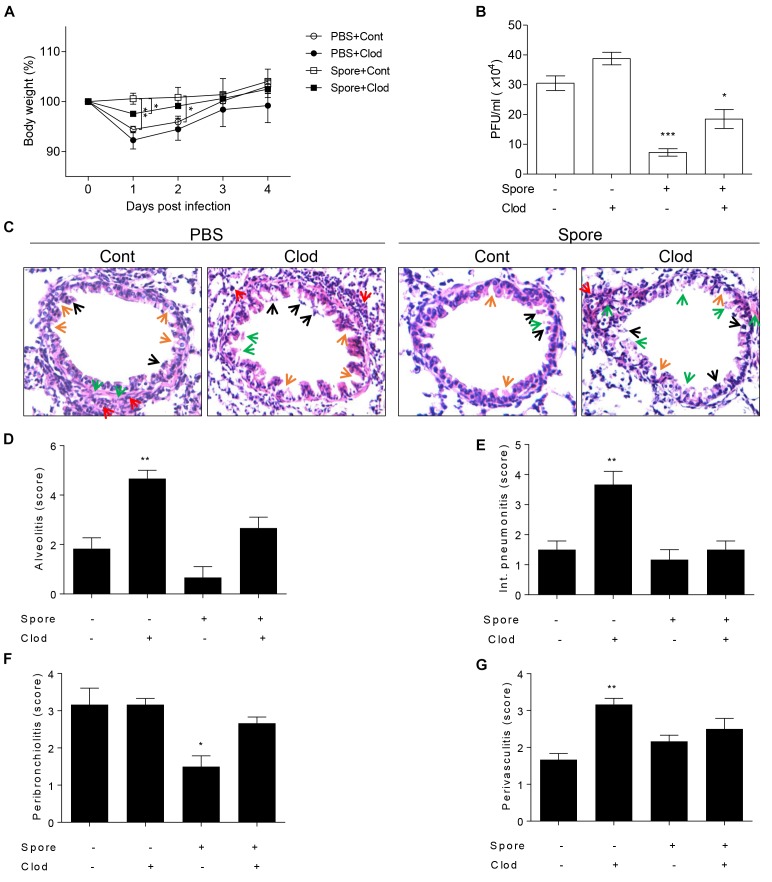
Alveolar macrophages in mice intranasally administered with spore play a key role in RSV infection. Mice were intranasally administered with spore at 5 days prior to RSV infection and injected with control or clodronate-encapsulated liposome through intratracheal on days 3 and 1 before the infection and sacrifice on 4 DPI. **(A)** Body weight was monitored daily after the infection and **(B)** viral load in the lung was examined at 4 DPI, respectively (*n* = 3). **(C)** At 4 DPI, perfused lungs were stained with H&E for histological examination by microscopy at 200 × magnifications and **(D–G)** scored for histopathology. Arrows indicated are as follows; orange, epithelium thickness and destruction; green, pulmonary edema; red, inflammatory cells; and black, cell death. Significant differences from results with the PBS control are ^∗^*P* < 0.05; ^∗∗^*P* < 0.01; and ^∗∗∗^*P* < 0.001, respectively.

To validate direct involvement of spore-activated AM for the protection from RSV infection *in vivo*, we depleted the AMs in spore pre-administered mice before RSV infection. As mentioned above, mice treated with spore did not have any significant weight loss (Figure 4A), in line with the ability of spore to clear the virus (Figure 4B). However, when we depleted AM by clodronate in spore-treated mice, they showed a moderate decrease of body weight (Figure 4A) and led to viral counts be increased in the lung (Figure 4B), suggesting that AM play an important role in the protection against RSV infection by *B. subtilis* spore treatment. The spore treatment can efficiently protect the epithelium and prevent infiltration of immune cells in alveolus (Figure 4C) and bronchi (Supplementary Figure S3), which is seen in the pathological score (Figure 4D–G) in support of the effect of AM. These data suggested that spore treatment prevents RSV infection efficiently, the effect is partially dependent on the AM. Taken together, these results support that the *B. subtilis* spore enhances host protection against RSV infection with support of AM.

### The Effects of Spore on Protection Against RSV Infection Are Dependent on MyD88 Signaling

**FIGURE 5:**
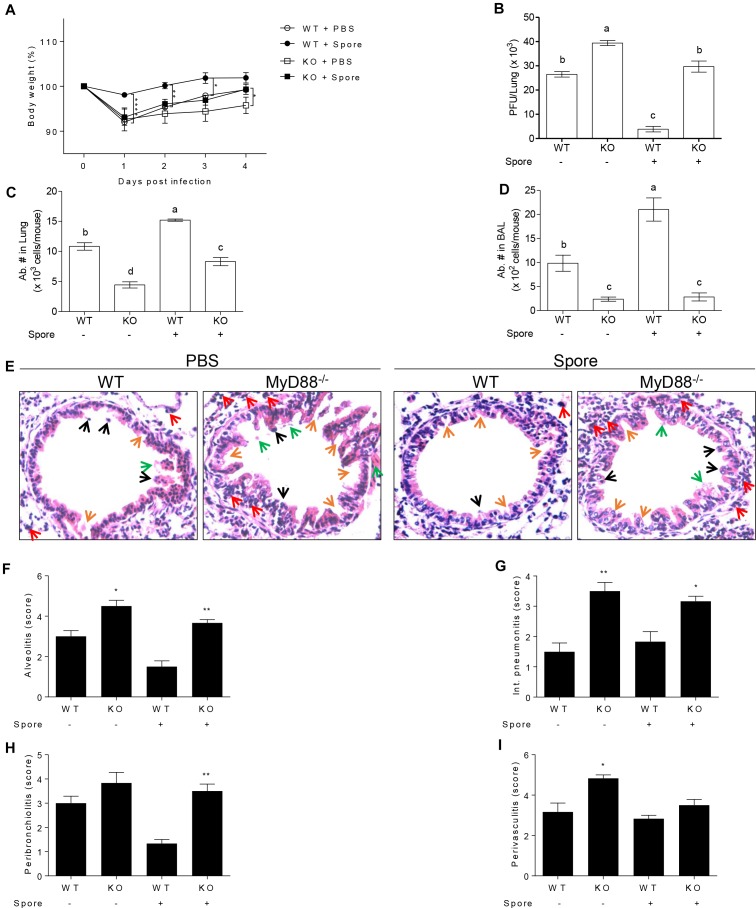
Protective mechanism of spore pre-treated mice infected with RSV is dependent on MyD88 signaling in AMs. Wild type or MyD88 knockout mice were administered i.n. with spore at 5 days prior to RSV infection. **(A)** Body weight was monitored daily after the infection (*n* = 3). At 4 DPI, **(B)** viral load in the lungs was analyzed by plaque assay and absolute number of AMs in **(C)** post lavaged lungs, and **(D)** BAL cells were acquired by flow cytometry. Values with different letters (a, b, c, d) are significantly different one from another (*P* < 0.05). **(E)** Blood-perfused lungs were stained with H&E for histological examination by microscopy at 200× magnification and **(F–I)** scored for histopathology at 4 DPI. Arrows indicated are as follow; orange, epithelium thickness and destruction; green, pulmonary edema; red, inflammatory cells; and black, cell death. Significant differences from results with the PBS control are ^∗^*P* < 0.05; ^∗∗^*P* < 0.01; and ^∗∗∗^*P* < 0.001, respectively.

The next question was which signaling pathways are involved in the capacity of spore to modulate anti-viral functions of AM. Since previous reports suggest TLR2 or 4 is in charge of viral infection as mentioned above, we presumed that spore might trigger MyD88 signaling upon direct engagement with TLRs on AM. In support of this idea, we used MyD88^-/-^ mice for RSV infection model with *B. subtilis* spore administration. MyD88^-/-^ mice were unable to induce protective response to RSV infection with reduced body weight (Figure 5A) and significantly high viral load (Figure 5B). More importantly, the administration of spore did not work on the recovery of MyD88^-/-^ mice at all. Consistent with these data, infiltration of AM in the lung (Figure 5C) and BAL (Figure 5D) was far less than wild-type mice and the inflammation with severe destruction of epithelium was also shown in alveolus (Figure 5E–I) and bronchi (Supplementary Figure S3) of MyD88^-/-^ mice even with *B. subtilis* spore treatment.

**FIGURE 6:**
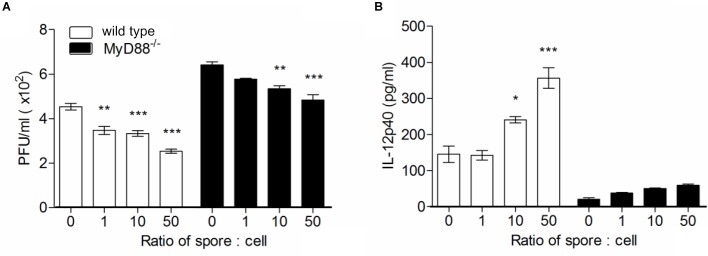
Bone marrow-derived macrophages pre-treated with spore develop antiviral effects through MyD88-dependent pathway. Wild type or MyD88 knockout BMMs were treated with spore for 24 h at ratio of spore per number of cells for 0, 1, 10, and 50. Then, the cells were washed and infected with 1 MOI virus for additional 12 h. **(A)** Viral titers from wild type or MyD88 knockout BMMs were measured by plaque assay and **(B)** the production of IL-12p40 was measured by ELISA. Data are expressed as mean ± S.E.M. for 3 independent experiments. ^∗^, ^∗∗^, and ^∗∗∗^ indicate significant differences at *P* < 0.05, *P* < 0.01, and *P* < 0.001, respectively.

To confirm the direct engagement of MyD88 signaling upon *B. subtilis* treatment in cellular level, we used bone marrow-derived macrophages (BMMs) from wild type or MyD88^-/-^ mouse and treated spore *in vitro* with RSV infection and conduct plaque assay with culture supernatant. In the same context with *in vivo* data, significantly higher viral loads were observed in MyD88-deficient BMMs compared to wild-type BMMs (Figure 6A). As expected, the capability of spore to reduce viral load was prominent in wild-type BMMs in a dose-dependent manner; unexpectedly, however, the effect of spore was, albeit less, also apparent even in MyD88-deficient BMMs. Consistent with data from viral loads, IL-12p40 production was markedly augmented by spore treatment and substantially higher in wild-type BMMs than MyD88-deficient BMMs (Figure 6B).

Taken together, these results suggest that *B. subtilis* spore can enhance the protective capacity against RSV infection affecting directly on the AMs, and these activation and M1-related effector cytokine production is dependent on MyD88 signal.

## Discussion

Importance of AM for host defense mechanism during early viral infection in respiratory tract is well established, however, the precise role of AM, especially in mice infected with RSV is less clear. In the present study, we focused on the specific role of AM treated with *B. subtilis* spore in RSV infected mice for their protection and brought the following four notable findings: (1) AM have a pivotal role for protection during the initial stage of RSV infection; (2) Administration of *B. subtilis* spore induces the activation of AM with up-regulation of GM-CSF and M1 macrophage-related cytokines; (3) The i.n. delivery of *B. subtilis* spore induces the increase of antiviral effector molecules against RSV infection; and (4) MyD88 signals in the AM are critical for the protection against RSV.

Induction of innate immune response in the early phase of viral infection is necessary for host immunity (McGill et al., 2009; Copenhaver et al., 2014), and thus over the years various attempts have been tried to improve the innate immunity in order to achieve the better protection against viral infection. Delivery of probiotics and their related materials is one of the strategies (Kiso et al., 2013; Yaqoob, 2014) and in the present study we sought a potential ability of spore to enhance innate immunity against RSV in line with other studies (Hong et al., 2008; McKenney et al., 2013).

It been reported that AM play an important function in diverse respiratory infections such as a primary recognition of antigens and scavenger to infected cells (Tate et al., 2010) and that the absence of AM-mediated defense mechanism led to the failure of protective immune responses, the exacerbation of pathology, and the accumulation of debris following pulmonary infection (Kolli et al., 2014) but the precise role and protective mechanism of AM in RSV infected mice are yet to be illuminated. Direct evidence showing important role of AM in the present study came from *in vivo* experiment with depletion of AM. The results showed that the AM-depleted mice suffered from high viral load and intense pathological episode as shown by the accumulation of dead cells and debris in bronchi and blood vessels, indicative of early pulmonary inflammation. All these findings clearly support the critical role of AM in controlling disease severity of mice infected with RSV. AM treated with *B. subtilis* spore rendered less susceptible to RSV infection and more importantly, anti-viral function of spore was markedly reduced upon depletion of AM, implying AM as a major target for *B. subtilis* spore as well as a key player for the protection.

How does then the spore influence on anti-viral function, differentiation, and/or migration of AM that would potentially benefit protective immunity to RSV? Our results showed that mice pre-treated spore through i.n. route rapidly up-regulated GM-CSF and M1 macrophage-related cytokines such as TNF-α, IFN-γ, IL-6, and IL-12p40. GM-CSF is a well-known essential factor for the differentiation (Guilliams et al., 2013), survival (Shibata et al., 2001), replenishment (Steinwede et al., 2011), and the ability of host defense (Ghoneim et al., 2013) of macrophages. Also, IFN-γ could lead monocytes differentiate into classically activated macrophages (M1-macrophages). This polarization is important in limiting tissue damage (Barros et al., 2013) and induces the increase of cytokines that enhance the ability of AM for the killing of intracellular pathogens (Martinez and Gordon, 2014). Thus, as a working mechanism for *B. subtilis* spore, it is conceivable that pre-treatment with *B. subtilis* spore might enhance the viability of macrophages, which promotes their antiviral functions. Alternatively, *B. subtilis* spore may facilitate AM proliferation and/or differentiation, skewing M1 vs. M2 balance toward favoring of M1 macrophage, which leads to an environment in which immune-stimulatory cytokines dominate for host protection against RSV. Supporting this idea, pre-treatment with spore indeed failed to induce TGF-β and PPAR-γ, which are known to induce differentiation of immune-suppressive M2 macrophages (Ginhoux, 2014; Schneider et al., 2014b). However, it is not always better to have M1 responses than M2 responses depending on how strong the immune response is and when the response was induced. It is important to induce appropriate and balanced immune responses because it has been reported that overwhelming pro-inflammatory responses with iNOS exacerbate the lung damage (Mgbemena et al., 2013). In addition, Th2 cytokines such as IL-4 and IL-13 induced after RSV infection could alleviate the damage of tissue (Shirey et al., 2010). Whether the preferences of M1/M2-type cytokines by spore indeed reflect AM proliferation/differentiation or selective enhancement of migration will need further investigation.

Besides the preference of M1 cytokines, mere increase of AM numbers we found after spore treatment would also have an advantage for protection against RSV infection, given that AM is known to play a role as reservoirs for inhaled antigens (Copenhaver et al., 2014) and thus the rapid increase of AM may effectively boost not only innate immunity but also prime adaptive immunity. Consistent with these, mice pre-treated with *B. subtilis* spore before RSV infection displayed a certain protective immunity as shown by significantly low level of viral titer and maintenance of body weight. Furthermore, *B. subtilis* spore treatment induced inflammatory monocytes with Ly6C high phenotype, known to regulate type I IFN signaling during acute viral pneumonia in mice (Seo et al., 2011) with an ability to differentiate into macrophages (Geissmann et al., 2010). Indeed, mice pre-treated with *B. subtilis* spore showed remarkably high IFN-β. Recent study showed that AM are the major source of type I IFNs during RSV infection and an underappreciated facet of type I IFNs-dependent resistance leads to a cell-extrinsic responses through rapid recruitment of antiviral inflammatory monocytes to the site of infection (Goritzka et al., 2015).

It would be important to postulate what are the mechanisms behind the modulatory ability of spore for AM. The most plausible explanation is that spore itself and/or its degraded components acts as a TLR ligand. TLR2 and TLR4 signaling pathways, in which MyD88 serves as a downstream molecule, are essential for the protection in RSV infection (Shirey et al., 2010). Consistent with those reports, in the present study, MyD88^-/-^ mice showed persistence of high viral counts in parallel with impaired body weight gain after the infection suggesting that MyD88 is indispensable for the protection against RSV infection. Furthermore, it was evident that MyD88^-/-^ mice failed to recruit AM into the lung region, irrespective of *B. subtilis* spore administration, suggesting that *B. subtilis* spore-induced increase of AM in the lung and BAL was dependent on MyD88.

MyD88^-/-^ mice also displayed severe pathological signs in the lung compared to wild type mice. These results imply that MyD88-dependent signaling might be the key mechanism by which AM treated with *B. subtilis* spore contribute to the protection of mice against RSV infection. This requirement of MyD88 signaling may account for the ability of spore to promote AM capable of producing GM-CSF and M1-related cytokines, which enhance their viability and M1 signature cytokines including IFN-γ and TNF-α, as well as producing IFN-β and IL-12p40, which may serve as an alarm signal and facilitate IFN-γ production. Whether all these MyD88-dependent effects by spore are indeed mediated through direct binding on TLR and whether there are MyD88-independent TLR signaling such as NOD2 being involved will need to be addressed. Collectively, our results provided a key modulatory role of *B. subtilis* spore in the anti-viral function of AM and have useful implication for effective treatment against RSV infection.

Even after depletion of AM, to a lesser extent, spore treatment can reduce the viral load of RSV. There are other possibilities than macrophages which may contribute to the protective function of spore against RSV infection. For instance, components derived from commensal microbes have been reported to induce anti-microbial peptides from epithelial cells that act as a first line defense against viral infection (Gomez and Prince, 2008). Furthermore, TLR2 signal can up-regulate the tight junction proteins in epithelial cells (Ragupathy et al., 2014) which can enhance the tolerance to viral infection.

Several studies have been done with *B. subtilis* spore to protect against infections in mice and human (Colenutt and Cutting, 2014; Lefevre et al., 2015, 2017). Even though the spores have been reported to be safe in human trials, safety should be more elucidated further, for example, on administration of spores in the respiratory tracts or as subcutaneous injection. In addition, the status of spore as a live or heat-killed form should be studied more in terms of safety. Collectively, we proved a key function of *B. subtilis* spore in antiviral activity and protection of lung environment against RSV-induced damage in mice infected with RSV via MyD88.

## Materials and Methods

### Mice and Ethics Statement

Female BALB/c mice, 6–8 week-old, were purchased from Orient Bio Inc., Korea. MyD88^-/-^ mice were purchased from Jackson laboratory (Bar harbor, ME, United States). Animals were maintained and procedures were performed with approval of the IACUCs of Seoul National University (Approval No.: SNU-130527-5) and International Vaccine Institute (Approval No.: 2012-022) in accordance to Laboratory Animal Act of Korean Ministry of Food and Drug Safety for enhancing the ethics and reliability on animal testing through appropriate administration of laboratory animals and animal testing.

### Preparation and Isolation of *B. subtilis* Spore

*Bacillus subtilis* was spread in an agar plate containing 3% Trypticase soy broth (TSB), 0.5% Yeast extract (YE), and 1.5% Bacto Agar (all from BD Biosciences, San Diego, CA, United States) and incubated at 37°C for 9 h. One colony was picked and inoculated in 25 ml of 3% TSB and 0.5% YE liquid media. Then, it was incubated for 5 h in the shaking incubator at 150 rpm at 37°C until the OD value reached between 0.45 and 0.6. For sporulation, culture was transferred to 500 ml of the autoclaved media containing 5 ml of 10% KCl, 5 ml of 1.2% MgSO_4_⋅7H_2_O (pH 7.6), 0.5 ml of 1 M Ca(NO_2_)_3_, 0.01 M MnCl_2_, and 1 mM FeSO_4_. The culture was incubated at 37°C for 48 h with shaking at 150 rpm. The cells were collected by centrifugation at 5516 × g for 10 min, re-suspended in distilled water, and incubated at 4°C for 48 h on the rocker. Then, the cells were sonicated at 35% amplitude (1 watt) for 90 s with 0.5 s pulse. Spore loaded on the layers of 35, 25, and 15% OptiPrep Density gradient (Sigma-Aldrich, Wt. Louis, MO, United States) was centrifuged at 10,000 × g for 40 min at 25°C without break for the purification. The spore was washed 3 times with distilled water and re-suspended in 1 ml of distilled water.

### Preparation and Isolation of Respiratory Syncytial Virus A2

Respiratory syncytial virus A2 strain was amplified as follows: HEp-2 cells (ATCC, Manassas, VA, United States) were grown in MEM containing 10% of FBS and 1% of antibiotics (Jang et al., 2011). When the cells reached at approximately 80% confluence, the cells were washed and inoculated with 0.01–0.05 MOI of virus in MEM containing 1% of antibiotics and 25 mM HEPES (Gibco, Grand Island, NY, United States). The cells were incubated for 2 h at 37°C, added MEM containing 6% of FBS only, and then incubated for additional 72–96 h. The cells were scraped and combined into the conical tubes on ice and centrifuged at 1,400 rpm for 4 min at 4°C. The cell pellets were collected into conical tubes, re-suspended with cold conditioned media with 60% sorbitol, sonicated for 15 min in slurry ice and centrifuged at 4,000–5,000 rpm for 10 min at 4°C, and then the sonication and centrifugation step was repeated under the same condition. The supernatants were transferred into new tubes, centrifuged at 23,000 rpm for 1 h at 4°C, and then discarded. The resulting whitish virus pellet was re-suspended with 500 μl of cold MEM and the titer was determined by standard RSV plaque assay.

### Isolation and Culture of Bone Marrow-Derived Macrophages and Macrophage Cell Line

Bone marrow was flushed from femurs and tibias of the mice using Dulbecco’s Modified Eagle Medium (DMEM, Gibco) supplemented with 2% FBS (Gibco). Red blood cells were lysed with ACK lysing buffer (Gibco) and whitish marrow cells were seeded in 90 × 15 mm Petri dish with complete media containing 10% FBS, 1% antibiotics with 20% L-929 conditioned media (Zhang et al., 2008) and cultured at 37°C in humidified incubator with 5% CO_2_ for 7 days. On day 3, another 5 ml of fresh complete media was added to each dish. On day 7, only adherent cells were collected using non-enzymatic cell dissociation solution (Gibco). MH-S cells (ATCC, Manassas, VA, United States), mouse AM cell line, were grown in RPMI-1640 GlutaMax medium containing 10% FBS, 1.5% antibiotics (all from Gibco) in a humidified incubator with 5% CO_2_ at 37°C.

### RNA Isolation and Quantitative Real Time-PCR

For quantitative analysis for the expression of GM-CSF, TNF-α, IFN-γ, IL-12p40, IL-6, PPAR-γ, and TGF-β1 at mRNA level, quantitative real time polymerase chain reaction (qRT-PCR) was conducted. RNA was extracted from perfused lungs using TRIZOL (Invitrogen, Carlsbad, CA, United States). Total RNA was isolated by adding chloroform followed by centrifugation at 4°C, 12,000 × g for 15 min and addition of isopropanol for 10 min at room temperature for RNA precipitation. RNA pellet obtained by washing with 75% ethanol was air dried for 10–15 min. Then it was re-suspended with DEPC distilled water (Sigma-Aldrich, Wt. Louis, MO, United States) and quantified with NanoDrop (Amersham Bioscience, United States) at A_260_. One microgram of RNA was reverse transcribed into cDNA and amplified with murine primers specific for GM-CSF (forward primer: 5′-CTGCCTTAAAGGGACCAAGAGA-3′, reverse primer: 5′-TTCCGCTGTCCAAGCTGAGT-3′), TNF-α (forward primer: 5′-GCCAACGGCATGGATCTC-3′, reverse primer: 5-GTGGGTGAGGAGCACGTAGTC-3′), IFN-γ (forward primer: 5′-GCCATCGGCTGACCTAGAGA-3′, reverse primer: 5′-GCAGTGTGTAGCGTTCATTGTCT-3′), IL-12p40 (forward primer: 5′-GAAAGGTGCGTTCCTCGTAGA-3′, reverse primer: 5′-GGAACACATGCCCACTTGCT-3′), IL-6 (forward primer: 5′-CACAGAGGATACCACTCCCAACA-3′, reverse primer: 5′-TCAGAATTGCCATTGCACAAC-3′), PPAR-γ (forward primer: 5′-CAGGAGCCTGTGAGACCAACA-3′, reverse primer: 5′-ATCAGTGGTTCACCGCTTCTTT-3′), TGF-β1 (forward primer: 5′-TCGTCTGCATTGCACTTATGC-3′, reverse primer: 5′-GTGGTGCCCTCTGAAATGAAA-3′), and GAPDH (forward primer: 5′-CTCCACTCACGGCAAATTCA-3′, reverse primer: 5′-GCCTCACCCCATTTGATGTT-3′). Real-time PCR was performed using Power SYBR Green PCR master mix (Applied Biosystem, Waltham, MA, United States) and analysis of the data was performed by One-step RT PCR (Applied Biosystem). Target gene expression was normalized to GAPDH expression.

### Measurement of Cytokine Production

Broncho-alveolar lavage (BAL) samples were collected via tracheotomy using 600 μl of PBS and the cells were separated from the BAL fluid by centrifugation at 1400 rpm for 5 min at 4°C. For *in vitro* experiments, supernatants from BMMs or MH-S after virus infection were carefully collected. Production of IFN-β (BioLegend, San Diego, CA, United States), IL-12p40, GM-CSF, and TNF-α (R&D System, Minneapolis, MN, United States) was examined using ELISA kit.

### Phenotypic Characterization of the Cells

To examine the absolute number of AM and other innate immune cells, BAL cells were collected as above and perfused lungs were isolated and minced through 70 μm cell strainer using MEM. The cells were stained with FITC-conjugated anti-CD11c (HL3), PE-conjugated anti-Siglec-F (E50-2440), PerCP-conjugated anti-Ly6C (AL-21), PE-Cy7-conjugated anti-Ly6G (1A8), APC-conjugated anti-CD11b (M1-70) or APC-conjugated anti-F4/80 (BM8), and APC-Cy7-conjugated anti-CD45 (30-F11) (all from BD Biosciences except anti-F4/80 from BioLegend). The cells were acquired using FACS LSR II and flow cytometric data were analyzed by using FlowJo software (Tree Star, San Carlos, CA, United States). We defined CD45^+^Ly6C^-^Ly6G^-^CD11c^+^Sigleg-F^+^F4/80^+^ as AMs, CD45^+^CD11b^+^Ly6C^+^Ly6G^-^ for inflammatory monocytes, and CD45^+^CD11b^+^Ly6G^+^Ly6C^-^ for neutrophils.

### Selective Depletion of Alveolar Macrophages

To selectively deplete AM, 350 mg per mouse of clodronate-encapsulated liposome (FormuMax Scientific Inc., CA, United States) was given i.t. in a volume of 50 μl at 1 and 3 days before the RSV challenge with or without spore treatment. To verify the depletion of AM, naïve mice were given control liposome via i.t.

### Virus Titration in the Lung

To determine the viral titers, lungs from RSV-infected mice were isolated at day 4 post-infection. The lungs were minced through 70-μm cell strainer using cold MEM. Cell lysates were collected and RSV titers were determined by plaque assay using HEp-2 cells. The virus titers in the whole lung were normalized to weight of the lung tissue and indicated as PFU/g.

### Lung Histology and Pathology Scoring

For histology studies, mice were administered with spore and/or clodronate encapsulated liposome prior to RSV infection. For control, mice were administered with PBS or control liposome. At 4 days post infection, BAL fluid was removed from lung and blood was perfused. Then, lungs were fixed with 4% paraformaldehyde and embedded in paraffin. Lung sections were produced and stained with Hematoxylin and Eosin to examine the abnormalities. Four inflammatory parameters were scored independently from 0 to 5 for each section: alveolitis (inflammatory cells within alveolar spaces), interstitial pneumonitis (increased thickness of alveolar walls associated with inflammatory cells), peribronchiolitis (inflammatory cells surrounding a bronchiole), and perivasculitis (inflammatory cells surrounding a blood vessel). Slides were randomized, read blindly, and scored for each parameter.

### *In vivo* or *in vitro* Spore Administration and RSV Infection

*In vivo* mouse model, each mouse was administered with either 1 × 10^9^ CFU of spore or, PBS as a control, i.n. in a volume of 20 μl at 5 days before the infection. Then, the mice were infected i.t. with 2 × 10^6^ PFU of live RSV A2. BMMs or MH-S cell line were cultured in 12-well plate (1 × 10^6^ cells/ml) with various ratio of spore for 24 h. Cells were washed with PBS and then infected with 1 MOI of live RSV A2 for additional 12 h.

### Statistical Analysis

Results are presented as means ± SEM. Statistical differences between two means were evaluated using unpaired Student *t* test. For comparisons of multiple groups, one-way ANOVA was used. Statistical significance was set at a *P* value of < 0.05.

## Author Contributions

C-HY conceived the study. MS, SH, and C-HY designed the study. JH, Y-CK, S-MP, and IC performed all the experiments. JH and C-HY wrote the manuscript. HC, B-CP, Y-MP, JC, and J-HC analyzed and discussed the data.

## Conflict of Interest Statement

The authors declare that the research was conducted in the absence of any commercial or financial relationships that could be construed as a potential conflict of interest.
