# Supplementary material for: Alveolar Macrophages Treated With Bacillus subtilis Spore Protect Mice Infected With Respiratory Syncytial Virus A2
Source: Front Microbiol. 2019 Mar 12;10:447. doi: 10.3389/fmicb.2019.00447 (PMC6423497; doi:10.3389/fmicb.2019.00447)
Supplement: Supplementary file 1 [file Data_Sheet_1.PDF]

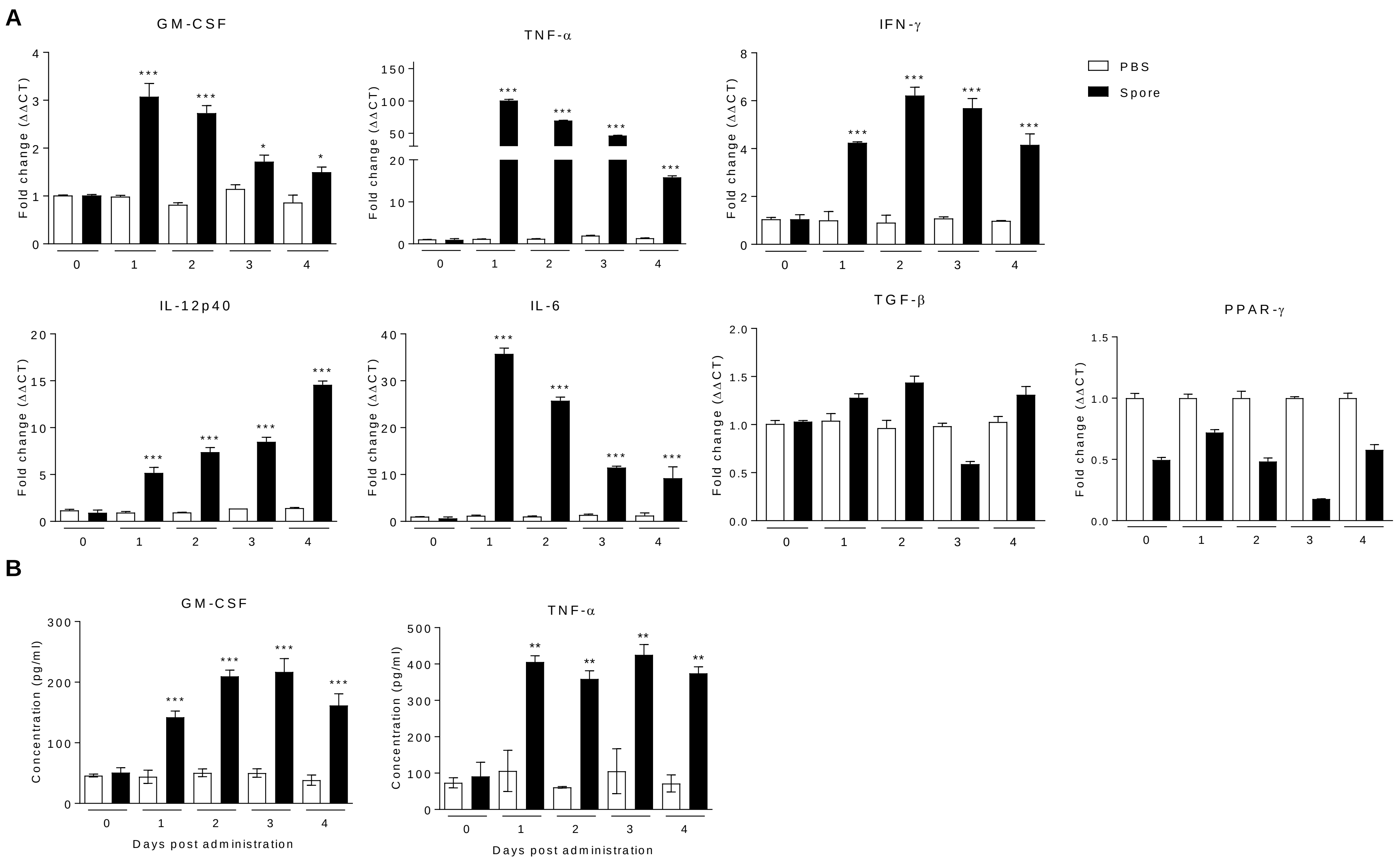

Fig. S1 Intranasal administration with spore induces differentiation of M1 alveolar macrophages and expression of GM-CSF. Mice were administered with spore via intranasal route and blood perfused lung was taken every day for 4 days. (A) Expression of genes associated with differentiation of M1(TNF-α, IFN-γ, IL-12p40 and IL-6) or M2(TGF-β) macrophages, GM-CSF, PPAR-γ, were analyzed by quantitative real-time PCR. (B) Protein expression levels of GM-CSF and TNF-α were measured by ELISA. Empty and filled bars indicate PBS and spore-treated mice, respectively. Data are presented as means ± S.E.M.(n=3). *,** and *** indicate significant differences at $P<0.05$, $P<0.01$ and $P<0.001$, respectively.

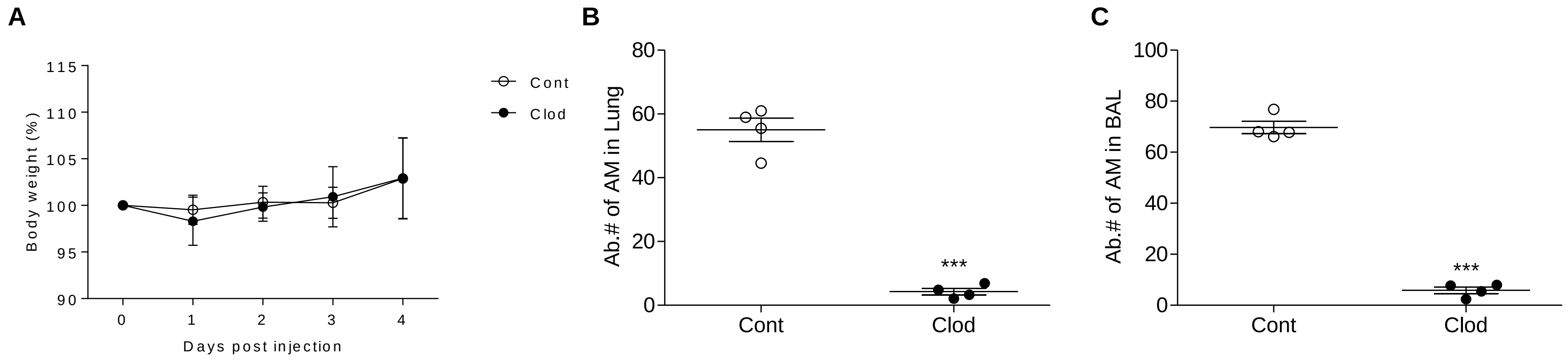

Fig. S2 Intratracheal injection of clodronate-encapsulated liposome leads to the efficient depletion of alveolar macrophages. Mice were injected with clodronate-encapsulated liposome via intratracheal route at day 1 and 3 before sacrifice. (A) Body weight was monitored daily after the injection, and absolute number of alveolar macrophages in the (B) lung and (C) BAL was analyzed at day 4 post administration (n=3). 'Cont' and 'Clod' indicate the mice injected with control liposome and clodronate-encapsulated liposome, respectively. Data are presented as means ± S.E.M.(n=3). *,** and *** indicate significant differences at $P<0.05$, $P<0.01$ and $P<0.001$, respectively.

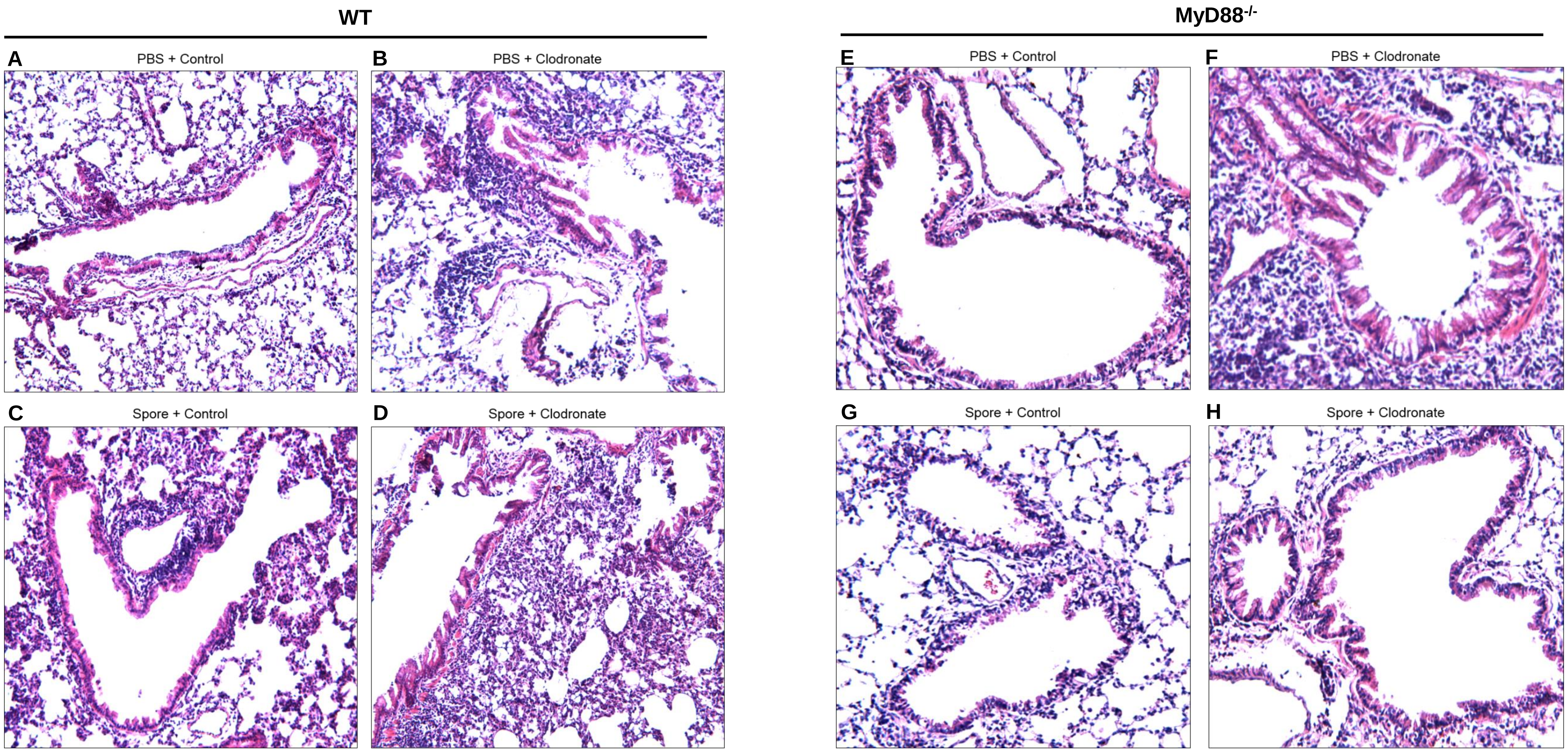

Fig. S3 Alveolar macrophages are indispensable for the protection in mice infected with RSV MyD88 signaling dependently. Mice were administered with spore via intranasal route 5 days prior to RSV infection. The mice were injected with control or clodronate-encapsulated liposome through intratracheal route 1 and 3 days before the infection. At DPI 4, perfused lungs were stained with H&E for histological examination by microscopy at 100X magnification. Bronchus and blood vessel of mice treated with (A) PBS / control-liposome, (B) PBS / clodronate-encapsulated liposome, (C) spore / control liposome, and (D) spore / clodronate-encapsulated liposome are shown. Wild type or MyD88 knockout mice were administered with spore via intranasal route 5 days before RSV infection. At DPI 4, perfused lungs were stained with H&E for histological examination by microscopy at 100 X magnification. Bronchus and blood vessel on the (E) PBS pre-treated wild type mice, (F) PBS pre-treated knockout mice, (G) spore pre-treated wild type mice, and (H) spore pre-treated knockout mice are shown.
